# Biodecolourisation of Reactive Red 120 as a Sole Carbon Source by a Bacterial Consortium—Toxicity Assessment and Statistical Optimisation

**DOI:** 10.3390/ijerph18052424

**Published:** 2021-03-02

**Authors:** Motharasan Manogaran, Nur Adeela Yasid, Ahmad Razi Othman, Baskaran Gunasekaran, Mohd Izuan Effendi Halmi, Mohd Yunus Abd Shukor

**Affiliations:** 1 Department of Biochemistry, Faculty of Biotechnology and Biomolecular Sciences, Universiti Putra Malaysia, Serdang 43400 UPM, Malaysia; haranz715@yahoo.com (M.M.); adeela@upm.edu.my (N.A.Y.); 2 Department of Chemical and Process Engineering, Faculty of Engineering and Built Environment, Universiti Kebangsaan Malaysia, Bangi 43600 UKM, Malaysia; ahmadrazi@ukm.edu.my; 3 Faculty of Applied Science, UCSI University, Kuala Lumpur 56000, Malaysia; baskaran@ucsiuniversity.edu.my; 4 Department of Land Management, Faculty of Agriculture, Universiti Putra Malaysia, Serdang 43400 UPM, Malaysia; m_izuaneffendi@upm.edu.my

**Keywords:** azo dyes, Reactive Red 120, decolourisation, phytotoxicity, optimisation, RSM

## Abstract

The application of microorganisms in azo dye remediation has gained significant attention, leading to various published studies reporting different methods for obtaining the best dye decolouriser. This paper investigates and compares the role of methods and media used in obtaining a bacterial consortium capable of decolourising azo dye as the sole carbon source, which is extremely rare to find. It was demonstrated that a prolonged acclimation under low substrate availability successfully isolated a novel consortium capable of utilising Reactive Red 120 dye as a sole carbon source in aerobic conditions. This consortium, known as JR3, consists of *Pseudomonas aeruginosa* strain MM01, *Enterobacter sp.* strain MM05 and *Serratia marcescens* strain MM06. Decolourised metabolites of consortium JR3 showed an improvement in mung bean’s seed germination and shoot and root length. One-factor-at-time optimisation characterisation showed maximal of 82.9% decolourisation at 0.7 g/L ammonium sulphate, pH 8, 35 °C, and RR120 concentrations of 200 ppm. Decolourisation modelling utilising response surface methodology (RSM) successfully improved decolourisation even more. RSM resulted in maximal decolourisation of 92.79% using 0.645 g/L ammonium sulphate, pH 8.29, 34.5 °C and 200 ppm RR120.

## 1. Introduction

Azo dyes account for 70% of the 9.9 million tons of industrial dye used annually, with a global turnover valued at USD 30.42 billion [1,2]. The continued demand for dyes and pigments causes an increase in the supply rate of 3.5% per annum. Most of the dyes synthesised contain azo compounds and are predominantly used in textile, paper, food, printing, cosmetic and leather industries [3]. These azo dyes are extensively used in fabric manufacturing due to low cost, ease of preparation, fastness, versatility and intensity of the colours [4]. Certain azo dyes contain chemical groups, which have a high affinity for metal ions [5]. These enhanced properties provide a high degree of chemical, biological and photocatalytic stability. Amongst azo dyes, Reactive Red 120 (RR120) is one of the widely used dyes in the textile industry due to it providing a high degree of chemical, biological and photocatalytic stabilities [1]. Being a diazo, RR120 is one of the hardest and most durable of reactive dyes and can resist degradation. Nevertheless, their resistance to breakdown due to time, exposure to sunlight, detergents, water and microorganisms results in poor degradation in the environment [6].

Pollution and poisoning by azo dyes still happen to this day. With an ever-increasing number of cancers, dye plays important role in contributing as a breakdown product producing toxic amines and benzene [7]. These toxic metabolics easily get into us through consuming water and fish exposed to dye waste. Discharge of untreated textile waste into nearby streams and river can cause anoxic conditions that are lethal to aquatic organisms [8]. Therefore, chemical treatment of the effluents is often employed to treat the waste. Chemical processes, such as the oxidative process, Fenton’s reagent, ozonation, photochemical, cucurbituril and electrochemical destruction, are examples used for decolourisation purposes [9]. This method is highly effective, faster, and most importantly can be done on large scale; but the drawback of this mechanism is quite distressing [9]. Some of these processes are dye-specific, making mix dye eluents take several processes to completely decolourise them. Moreover, the application of such technologies is usually applied by high-end industrial producers and is limited to small scale manufacturers due to the cost of handling and maintenance. Therefore, the removal of azo dyes requires an alternative cheap and environmentally friendly approach whereby the role of bacteria could provide such.

The use of microorganisms in treating dye wastes is one of the most favourable processes in comparison to other applications due to its practicality, productivity, simplicity, and inexpensiveness [10]. Bioremediation allows the bacteria to consume the available dye compound during cell propagation. Unlike biosorption, even though the dyes have been removed from the wastewater, the absorbate still holds the dye compounds which requires further physio-chemical treatment to reduce it, whereby decolourisation using bacteria solves both problems by eliminating the dye compound and reducing the need for chemical exposure [11]. Various azo dye decolourisation using bacteria, such as Reactive Black 5 [12], Black 5 [13], Methyl Orange [14], Acid Red [15], and Green 19 [16], have been reported before.

For over a century, microbial decolourisation of azo dyes has been an unsolved puzzle among scientists. Previously, in microbial azo dye decolourisation, the focus was centred on isolating bacteria or consortia that have a higher azo dye tolerance level needed as a tool for bioremediation [17,18]. To achieve this, various additional carbon sources and a higher concentration of co-substrate were introduced. During the last ten years, the attention has shifted towards isolating microorganisms with the ability to decolourise azo dyes with complete mineralisation ability [14]. The focus changed mainly because degraded metabolites of azo dye were found to be toxic [19].

Improper cleavage of azo dyes during the decolourisation process could result in toxic metabolites that are far more toxic than the parent compound. As such, these compounds generally consist of benzene and aniline groups, which are a precursor to carcinogenic and mutagenic effects [19]. Even though decolourisation has occurred, there is always a chance that colourless metabolites produced by the bacteria end up being toxic. A race to find the best strains to solve azo dye pollution ended up manifesting more problems rather than providing a good solution. This dilemma has been going on for the past few decades, leading to various methods and media used for the purpose of isolation. However, the role of method and its influence on media used for azo dye decolourisation has not been compared nor studied before. Having this understating could provide crucial data needed to better isolate azo dye consuming bacteria. Since each media composition is different to the others, the role of media in supporting azo dye decolourisation needs to be elucidated.

In this study, dye contaminated samples from the heavily polluted industrial waste site were obtained from Juru River in Malaysia. These samples were examined using a different method of isolation to identify bacterial strains able to decolourise RR120 as sole carbon source. The role of different media compositions and co-substrates in sustaining maximum RR120 decolourisation was also further investigated. The toxic level of the produced metabolites was assessed using mung beans. Since decolourisation of azo dye involving diazo as a sole carbon source is a slow process, factors affecting decolourisation were optimised to reinforce the process of degradation. Further optimisation was done using a surface methodology approach (RSM) which utilised a mathematical and statistical approach to develop and study the interaction between two factors. To the best of our knowledge, this is the first isolation of aerobic decolourisation of RR120 by a novel bacterial consortium.

## 2. Materials and Methods

### 2.1. Chemicals, Reagents, and Equipment

The Reactive Red 120 (RR120) used in this research was purchased from Sigma, Aldrich, CO, USA. Nutrient agar, yeast extracts and nutrient broth were obtained from Friedemann Schmidt Shd. Bhd. Malaysia. Tris and acetate buffers were obtained from Merck KGaA, Germany. Meanwhile, other chemicals used in this study were obtained from Fisher Malaysia.

### 2.2. Sample Soil and Water

Juru River was chosen as a prime location for sampling as the state contributed 28.2% of total water pollution originating from the textile industry [20]. Most of these industrial factories are located near to Juru Riverside. Industrial textile wastewater effluent was collected in a 20 mL sterilised falcon tube and samples were brought to the lab and processed within 24 h. For sample preparation, 10 uL cycloheximide was introduced to prevent fungal growth. The locations of samples collected were recorded using the map coordinates provided by Google Earth to locate the exact locations from which the samples were collected (Table 1)

### 2.3. Screening for RR120 Dye Decolourising Bacteria

The study was initiated by studying the best for in obtaining bacteria capable of decolourising Reactive Red 120 as a sole carbon source.

#### 2.3.1. Method 1: Isolation Using Nutrient Broth

Method number 1 is commonly used to rapidly isolate dye decolourising bacteria. This involves samples inoculated in nutrient broth containing some sort of azo dye [21,22,23]. Briefly, a 1 mL of sample was inoculated in 100 mL nutrient broth containing 100 ppm filter-sterilised RR120 dye. The culture was incubated in a 250 mL conical flask at room temperature under static conditions for 72. After 72 h of incubation, the sample was streaked in nutrient agar containing 100 ppm RR120. The morphologically distinct bacterial isolates showing a clear zone of decolourisation were selected for identification.

#### 2.3.2. Method 2: Isolation through a Straightforward Process

Method 2 is a straightforward method for isolating dye decolourising bacteria without the process of acclimatisation. This was done by inoculating 1 mL sample in minimal salt medium (MSM) containing 100 ppm RR120 [24,25]. The composition used MSM in g/L of dH_2_O was pH (7.0 to 7.2) yeast extract (1.0) Na_2_HPO_4_ (1.0), KH_2_PO_4_ (1.0), NaCl (1.0), MgSO_4_.7H_2_O (0.5), CaCl_2_ (0.01) [26]. A 1 mL sample was incubated in 100 mL sterilised MSM in a 250 mL conical flask containing 100 ppm filter-sterilised RR120. The culture medium was incubated for 72 h in shaking condition at 31 °C. The dye decolourising strain was isolated using streaking on MSM agar containing 100 ppm RR120, which showed a clear zone of decolourisation.

#### 2.3.3. Method 3: Isolation through Long Acclimatisation Process

Method 3 involves the acclimation of bacterial strains to improve decolourisation capacity by slowly introducing an increased concentration of RR120 while reducing substrate and carbon availability [27,28]. A 1 mL sample was first inoculated in nutrient broth containing 25 ppm RR120 and was incubated at 31 °C for 72 h in shaking conditions (150 rpm). A 1 mL of sample was taken from a previously grown culture and incubated in another 100 mL of MSM-containing 8 g/L glucose, 3 g/L yeast extract and 25 ppm RR120. The inoculated medium was incubated at 31 °C under shaking conditions (150 rpm) for a week. From this suspension, 1 mL was transferred into fresh MSM-containing 5 g/L glucose and 3 g/L yeast extract with 50 ppm RR120. A similar successive transfer was done with 2 g/L glucose, 3 g/L yeast extract and 100 ppm RR120. The next successive transfer was done with 3 g/L yeast extract and 150 ppm RR120 followed by 1 g/L yeast extract and 200 ppm RR120. The final successive transfer was done with MSM containing 200 ppm RR120 only. For each successive, the culture medium was incubated at 31 °C for a week in shaking conditions. Those bacterial strains that grew on MSM (no glucose and yeast extract added) containing 200 ppm RR120 as the sole carbon source was selected for further identification.

### 2.4. Analytical Method

Decolourisation of Reactive Red 120 was determined by colourimetric determination. The absorbance of each solution was determined using a 525 nm wavelength against blank using a Shimadzu U.V. Mini 1240 spectrophotometer. Meanwhile, bacterial growth was measured using a colony-forming unit per millilitre technique (CFU/mL) and optical density (OD) at 600 nm [21].

$$\%Decolourisation=\frac{Initial\;absorbance-final\;absorbance}{Initial\;absorbance}\times 100.$$

### 2.5. Identification of Isolates

The biochemical test was done to determine the bacteria species based on physiological differences. The included test was Gram staining, oxidase/catalase test, indole and nitrate production, citrate, Voges-Proskauer, urease and sugar test. The genomic DNA of the selected strains were extracted with the innuPREP extraction kit (Analytik Jena GmbH, Jena, Germany), following the manufacturer’s recommended procedure. The Polymerase chain reaction (PCR) product was produced using a Biometra T-Gradient Thermocycler. The mixture contains 1 µL Template DNA, 14.2 µL dH_2_O, 1.2 µL of 25 mM MgCl_2_, 2 µL of 10 × Taq polymerase buffer, 0.5 µL of 10 mM Deoxynucleotide triphosphate mix, 0.5 µL of 10 µM forward primer, 0.5 µL of 10 µM reverse primer, and 0.1 µL Taq DNA polymerase. PCR universal primer; 27F: 5′-AGA GTT TGA TCC TGG CTC AG-3′ and 1492R: 5′-TAC GGT TAC CTT GTT ACG ACT T-3′, corresponding to forward and reverse primers of 16S rRNA, was used to amplify the 16S rRNA gene of the isolates. The thermal cycler protocol was comprised of an initial 4 min denaturation at 95 °C for 1 cycle, followed by 30 cycles of 1 min denaturation at 95 °C, primer hybridisation at 52 °C for 1 min and elongation at 72 °C for 1 min with 1 final cycle for a 7 min extension step at 72 °C. Successful PCR product was purified using a GeneJET Gel Extraction and DNA Cleanup Kit (Thermo Scientific, Waltham, MA, USA) before a sequencing process using an ABI 3730xl DNA Analyzer (Applied Biosystems, Foster City, CA, USA).

### 2.6. Phylogenetic Analysis

The top 20 16s rRNA sequence with the highest similarity of the related isolate’s sequence was obtained from GenBank using BLAST (Basic Local Alignment Search Tools) (www.ncbi.nlm.nhi.gov/BLAST/bl2seq/, accessed on 10 August 2020). Using Clustal Omega program (www.ebi.ac.uk/Tools/msa/clustalo/, accessed on 10 August 2020), the 20 sequence from the GenBank was aligned with a target isolate’s gene. The obtained aligned files were analysed using evolutionary analysis software PHYLIP v 3.6. The neighbour-joining method was used to infer evolutionary history [29]. *Bacillus cereus* ATCC 14,579 (Accession no. MG708176) was used as an out-group to construct the phylogenetic tree.

### 2.7. Media Selection for Optimal RR120 Decolourisation

The effect of media composition on the obtained consortium for RR120 decolourisation was investigated. Five media with different composition was used for the best media selection (Table 2). Each consortium was inoculated in MSM containing filter-sterilised 50 ppm RR120 and incubated at room temperature in shaking conditions (150 rpm) at 31 °C. The shaking condition was done regardless of the original media condition to prevent anaerobic decolourisation. Resting cells were used to remove any nutrient residue from previously cultured medium that could influence dye decolourisation. Reading of dye decolourisation was taken at 24 h.

### 2.8. Phytotoxicity Study Using Vigna Radiata

The phytotoxicity study was carried out using mung bean (*Vigna radiata*). All the mung bean seeds were first sterilised using 70% ethanol for 3 min followed by 5% sodium hypochlorite for another 3 min [35]. Then those sterilised mung beans were washed 5 times with sterilised dH_2_O and soaked for 3 min in sterilised dH_2_O [36]. Decolourised culture medium on the 24 h was centrifuged at 10,000× *g* for 30 min at 4 °C and the resulting supernatant was filter-sterilised using a 0.22 μm pore filter. The sample was given 3 mL of filter sterilised untreated/treated sample per day. The control set was carried out using distilled water at the same time. The germination (%) and length of shoot and root was recorded after day 7.

### 2.9. Effect of Co-Substrate on Decolourisation of RR120

The role of co-substrate in improving the decolourisation activity of the obtained consortium was investigated in this study. The purpose of the experiment was to determine the ability of said bacteria to decolourise RR120 without any available co-substrate. This was done by removing any glucose and yeast extract from the media composition based on Table 2.

### 2.10. Effect of Yeast Extract on RR120 Decolourisation

Decolourisation without any presence of co-substrate is a relatively slow process and rare. Therefore, to enhance the decolourisation azo dye, the yeast extract is supplemented as a co-substrate. The yeast concentration of 0 g/L to 5 g/L with an interval of 0.5 g/L was supplemented into 100 mL of the best MSM in sustaining decolourisation of RR120 as the sole carbon source.

### 2.11. Optimisation of RR120 Decolourisation Using One-Factor-At-A-Time

The best medium in sustaining in maximum RR120 decolourisation based on Table 2 and its condition was further optimised in this study. Four different parameters, such as nitrogen source and concentration, pH, temperature, and dye concentration, were used to study the growth and decolourisation rates. Five different nitrogen sources were used in the experiment—0.5 g/L each of ammonia chloride (NH_4_Cl), ammonium sulphate (NH_4_SO_4_), potassium nitrate (KNO_3_), urea (CH_4_N_2_O) and magnesium nitrate (Mg(NO_3_)_2_). A pH ranging from 5 to 9 was optimised using appropriate buffers. The varied pH was adjusted using different overlapping buffers systems whereby at pH 5.0 and 6.0, 50 mM a citric buffer was used, while for pH 6.0, 7.0 and 8.0, 50 mM, phosphate buffer was utilised. For pH 8.0 and 9.0, 50 mM, Tris buffer was used. To study the effect of temperature on RR120 decolourisation, temperature ranging from 20 to 50 °C were used. The ability of bacteria to degrade a high concentration of RR120 was studied to ascertain the optimum concentration of RR120. RR120 concentrations of 25, 50, 100, 150, 200, 300, 400, and 500 ppm.

### 2.12. Optimisation of RR120 Decolourisation Using Response Surface Methodology

Statistical optimisation using RSM was carried out based on one-factor-at-a-time (OFAT) factors range levels. A Design Expert v 13.0 (Trial Version, Stat- Ease Inc., Minneapolis, MN, USA) was utilised to optimise the factors. Two stages of optimisation were done using response surface methodology to understand the interaction between factors involved in improving RR120 decolourisation by consortium JR3. Significant factors were screened using Plackett-Burman and further optimisation was done using a central composite design.

#### 2.12.1. Statistical Optimisation Using Plackett-Burman and Central Composite Design

The Plackett-Burman design was chosen to define the most appropriate parameters among the different factors. Design Expert v 13.0 was used for this experiment, along with four parameters selected for a total of 12 experimental runs. Each parameter was analysed at two different levels (low and high) as shown in Table 3. After a variance analysis was conducted, factors with *p* < 0.05 were considered significant and were further optimized using a central composite design.

#### 2.12.2. Optimisation of Significant Factors Using a Central Composite Design

A central composite design was used to analyse the optimal RR120 decolourisation by consortium JR3 and the interaction among the factors with 30 experimental runs. The levels of four significant factors and their levels are illustrated in Table 4. Based on the conformation run resulting from central composite design (CCD) analysis, an experiment was done to assess the best condition for RR120 decolourisation by consortium JR3.

### 2.13. Statistical Analysis

All the experiments were conducted in triplicate. Error bars are used to show experimental errors (standard deviation of three determinations). Data from the experiments were statistically analysed using GraphPad v3.5 (GraphPad Software Inc, San Diego, CA, USA) and one-way analysis of variance was done using Tukey’s test.

## 3. Results

### 3.1. Determination of Reactive Red 120 Absorbance Pattern

**Figure 1:**
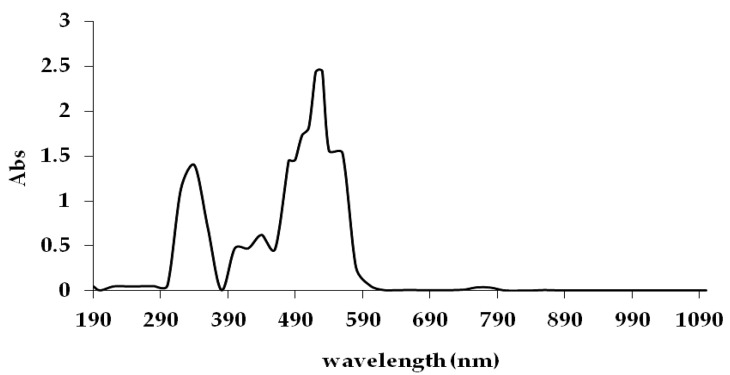
Scanning absorption spectrum of Reactive Red 120 at different wavelength (nm). Maximum absorption was obtained at 525 nm.

Figure 1 shows the maximum absorbance of RR120 based on the absorption spectrum at a range of 510 to 550 nm, where the highest peak was at 525 nm. This result is in agreement with the previously reported RR120 maximum absorbance, which is within the range of 525 ± 10 nm [26,30]. Hence, the decolourisation of RR120 was measured at 525 nm against the blank.

### 3.2. Isolation of RR120 Decolourising Bacteria

All three methods result in three different consortia. They are, namely, consortium JR1 based on method 1, consortium JR2 based on method 2, and consortium JR3 based on method 3. Initial screening results in 4 isolates (JR1-1, JR1-2, JR1-3 and JR1-4) from consortium JR1, 2 isolates (JR2-1 and JR2-2) from consortium JR2 and 3 isolates (JR3-1. JR3-2 and JR3-3) from consortium JR3. Method 1 yields a higher number of isolates followed by method 3 and then method 2. All this isolate shows the ability to either decolourise RR120 or simply to be able to proliferate under the presence of RR120. For now, all these isolates have been clustered together to form a consortium based on the method of isolation. Since the bacterium was isolated from a single consortium, the role of mutual symbiosis between the bacteria might have a positive impact on the decolourisation rate of RR120.

### 3.3. Identification of Consortium JR1, JR2 and JR3

**Figure 2:**
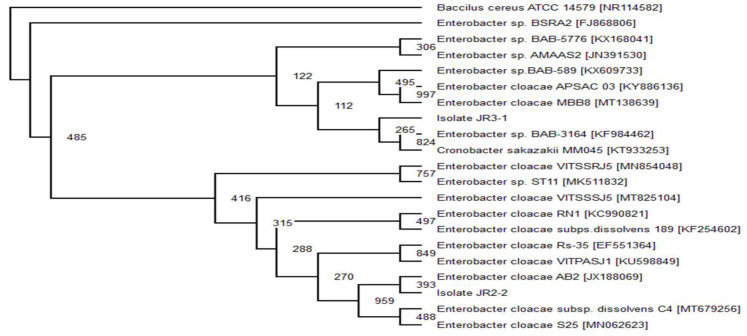
The cladogram was carried our based-on neighbour-joining technique illustrating the phylogenetic relationship between isolate JR2-2 and JR3-1 with other interconnected analogous bacterium based on 16S rRNA gene sequence examination.

The results from the bootstrap analysis of 16S rRNA are consistent with the biochemical properties and morphological results (Table 5). The phylogenetic analysis of isolate JR2-2 and JR3-1 illustrated its similarity with the genus of *Enterobacter* species, which were constructed with the foremost 20 sequences, which showed at least 98% in sequence identity (Figure 2). Isolate JR2-2 showed a sequence similarity of 393 with *Enterobacter cloacae* AB2 [JX188069]. Where else, isolate JR3-1 did not closely match with any known subspecies, thus it was identified as *Enterobacter* sp. MM05.

**Figure 3:**
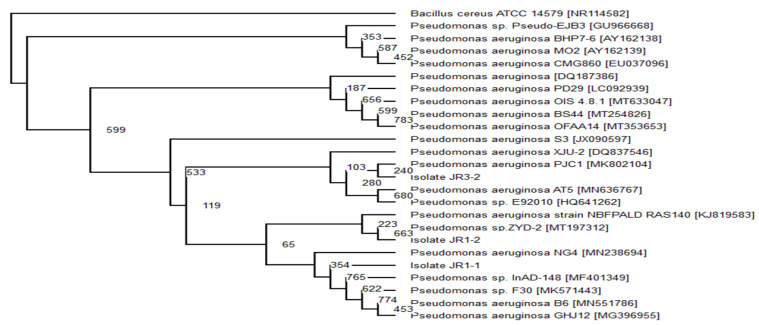
The cladogram was carried out based-on neighbour-joining technique illustrating the phylogenetic relationship among isolate JR1-1, JR1-2 and JR3-2 with other interconnected analogous bacterium based on 16S rRNA gene sequence examination. *Bacillus cereus* was the outgroup.

Isolate JR3-2 showed a strong bootstrap value associated with *Pseudomonas aeruginosa* PJCI [MK802104], which was distinct from other *Pseudomonas* species, therefore it was identified and registered as *Pseudomonas aeruginosa* MM01 (Figure 3). However, both isolates, JR1-1 and JR1-2, are not in the clade with other subspecies, therefore both are registered as *Pseudomonas* sp. MM02 and MM03, respectively.

**Figure 4:**
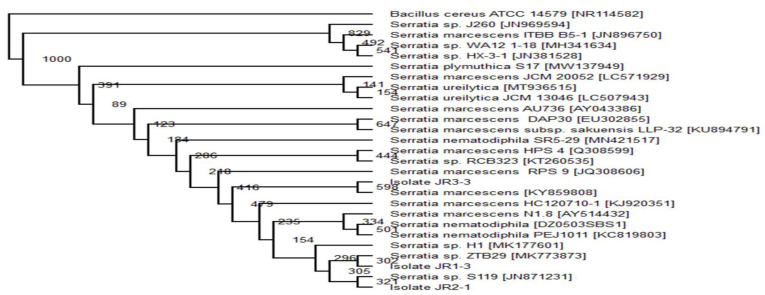
The cladogram was carried our based-on neighbour-joining technique illustrating the phylogenetic relationship among isolate JR1-1, JR1-2 and JR3-2 with other interconnected analogous bacterium based on 16S rRNA gene sequence examination. *Bacillus cereus* was the outgroup.

**Figure 5:**
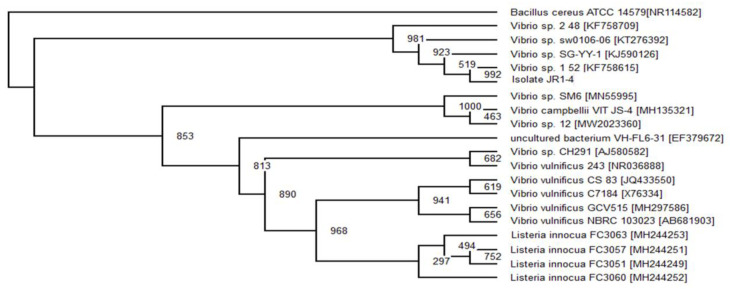
The cladogram was carried our based-on neighbour-joining technique illustrating the phylogenetic relationship of isolate JR1-4 with other interconnected analogous bacterium based on 16S rRNA gene sequence examination. The species names are trailed by the accession numbers of their 16S rRNA. *Bacillus cereus* was the outgroup.

Meanwhile, isolates JR1-3, JR2-1 and JR3-3 illustrated a similarity with the genus *Serratia* species, which was constructed from 20 sequences highly analogous in identity. Isolate JR1-3 was identified as *Serratia* sp. MM07 and isolate JR2-1 as *Serratia* sp. MM08, due to both strains having close similarity to *Serratia* sp. ZTB29 [MK773873] and *Serratia* sp. S119 [JN871231], respectively (Figure 4). Based on the bootstrap value, isolate JR3-3 showed sequence similarity with a value of 598 to *Serratia marcescens* [KY859808], thus, it was registered under GenBank as *Serratia marcescens* strain MM06 (Table 6). In all three obtained consortia, only one isolate from Consortium JR1 was identified as *Vibrio* sp. MM09, which was previously labelled as isolate JR1-4 (Figure 5).

### 3.4. Effect of Media Composition on RR120 Decolourisation by Consortium JR1. JR2 and JR3

**Figure 6:**
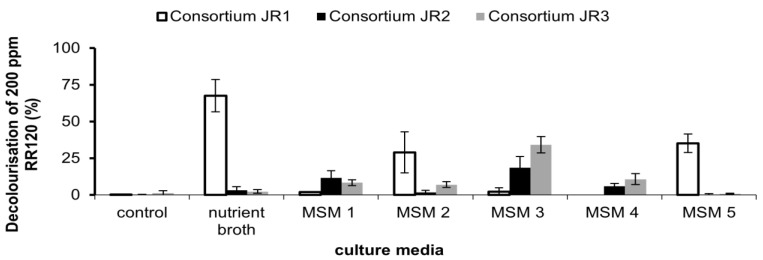
Effect of different media composition on decolourisation of 50 ppm Reactive Red 120 by consortium JR1, JR2 and JR3. Data represent mean ± SD, *n* = 3.

The nutrient broth compositions of MSM 2 and MSM 5 resulted in higher decolourisations of RR120 in consortium JR1 compared to the rest of the media (Figure 6). Meanwhile, for consortium JR2, MSM 1 and MSM 3 resulted in the best media for RR120 decolourisation. Consortium JR3, which was isolated using method 3, illustrated the best decolourisation ability in MSM 3 and MSM 4. The method of isolations showed different affinity towards the media being used. For instance, using the isolation of method 1, the obtained consortium JR1 showed a better decolourisation in media containing nutrient broth, glucose, and high concentration of yeast extract. Direct isolation using NA containing RR120 only results in a bacterium that is able to decolourise RR120 in the presence of a carbon source. Consortium JR1 was unable to grow and decolourise RR120 in MSM 4 as it contains no alternate carbon source. Less than 10% decolourisation was observed in the media containing lower than 3 g/L of yeast extract in MSM 1 and MSM 3.

### 3.5. Effect of RR120 and Decolourised Metabolites on Vigna Radiata

The relative sensitivity of *Vigna radiata* against treated and untreated RR120 is listed in Table 7. The untreated RR120 at 50 ppm concentration resulting in 63.3% inhibition of germination was *Vigna radiata*. The root and shoot lengths were reduced to 4.23 and 7.76 cm, respectively. Only consortia JR2 and JR3 showed improvement in seed germinations, and root and shoot lengths when treated with decolourised samples in all 6 mediums. As shown in Table 5. JR3 + MSM1, JR3 + MSM2 and JR3 + MSM4 variants significantly (*p* < 0.005) reduced phytotoxicity of 50 ppm RR120 compared to untreated samples. Consortium JR2 was the second best as it improved seed germination with an average of 66.21% in all 6 media. MSM4 and MSM5 performed much better in terms of aiding RR120 removal with probably fewer toxic metabolites produced than in consortia JR3 and JR2. This illustrates that the resulting metabolites from the treated sample were safe and the toxicity of RR120 had been alleviated. However, when treated with decolourised samples of JR1, a significant reduction in all three parameters was observed. Even though JR1 reduces RR120 by 67.6% in the NB medium, the decolourised metabolite did not improve the plant conditions but further worsened it compared to RR120 alone. In comparison with all three consortia, only consortium JR3 proved to have a better decolourisation ability than RR120, and the resulting metabolite was much safer.

### 3.6. Effect of Co-Substrate on the Decolourisation of RR120 by Consortium JR3

**Figure 7:**
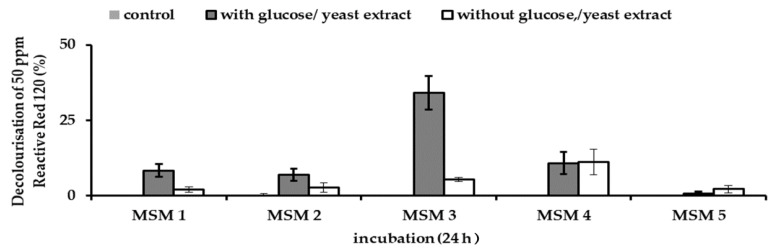
Effect of glucose and yeast extract in the media composition on the decolourisation of 50 ppm Reactive Red 120 by consortium JR3 at 24 h. Data represent mean ± SD, *n* = 3.

When glucose and yeast extract were removed from the media composition, the decolourisation of Reactive Red 120 by consortium JR3 was significantly reduced (Figure 7). This shows that the decolourisation of RR120 was heavily dependent on the presence of additional carbon or co-substrate availability. The highest decolourisation was observed in MSM 4. Decolourisation was significantly reduced from 34.2% to a mere 5.2% in MSM 3, when yeast extract was removed from the media composition. This illustrates that MSM 4 can sustain higher RR120 decolourisation in consortium JR3. Unlike MSM 1, MSM 2, MSM 3, and MSM 5, only MSM 4 contained trace elements. Trace elements are important macro and micronutrients. As these data strongly support the presence of trace elements in enhancing azo dye decolourisation, even when no co-substrate was present, therefore, MSM 4 was chosen as the best media in aiding RR120 decolourisation by consortium JR3.

### 3.7. Effect of Yeast Extract on RR120 Decolourisation

**Figure 8:**
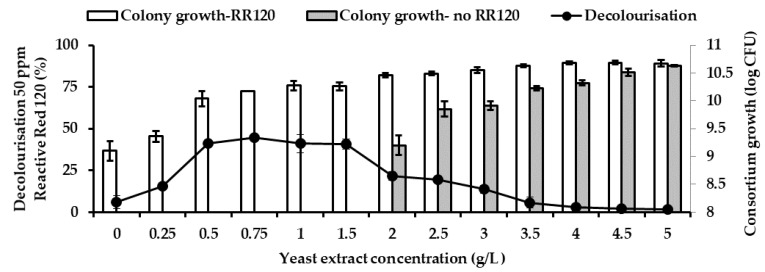
Effect of different yeast extract concentration on consortium JR3′s ability to decolourise 50 ppm Reactive Red 120. Data represent mean ± SD, *n* = 3.

The effects of different yeast extract concentrations on the decolourisation of 50 ppm RR120 and colony growth by consortium JR3 in MSM 4 is depicted in Figure 8. Increasing yeast extract concentration resulted in improved in decolourisation of RR120 and colony growth till 1.5 g/L. Beyond 1.5 g/L showed a decrease in the decolourisation of RR120. Highest RR120 removal was observed at 0.75 g/L with 44.5% and colony growth of 10.18 log colony-forming unit (CFU), followed by 0.5 g/L with 41.1% decolourisation and colony growth of 9.97 log CFU. Based on ANOVA, there were no significant differences (*p* > 0.05) between 0.5 g/L with 0.75 g/L, 1 g/L, 1.5 g/L yeast extract in term of decolourisation and colony growth, suggesting similar results were obtainable at those concentrations. Decolourisation remains low at 0 g/L with 4.9% and colony growth of 9.1 log CFU. However, significant improvement was observed at 0.25 g/L with 15.5% decolourisation illustrating that the presence of yeast extract improves RR120 decolourisation rate. However, an increase beyond 1.5 g/L dramatically reduces decolourisation of RR120 despite colony growth remaining high. To understand the effect, consortium JR3 was exposed to different concentrations of yeast extract without the presence of RR120. It was found that, without RR120, consortium, JR3 was only able to survive with 2 g/L yeast extract onwards. This illustrates that there is enough carbon source from yeast extract to sustain the consortium JR3 growth at 2 g/L.

### 3.8. Effects of Different Nitrogen Sources on Decolourisation of RR120

**Figure 9:**
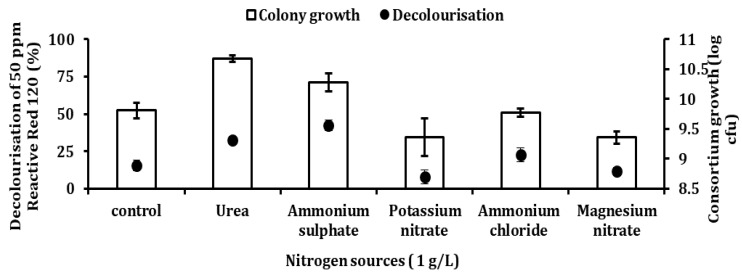
Effects of different nitrogen source type on the decolourisation of 50 ppm RR120 by consortium JR3. Data represent mean ± SD, *n* = 3.

Figure 9 shows the effect of different types of nitrogen sources on the decolourisation of 50 ppm RR120 and bacterial growth by consortium JR3. Ammonium sulphate aided the highest decolourisation of 42.5% with colony growth of 10.23 log CFU. Urea followed second with a decolourisation of 31.8% and a colony growth of 10.67 log CFU. Meanwhile, ammonium chloride showed a decolourisation of 22.17%, followed by control with 15.4%. Both ammonium chloride and control demonstrated no significant difference (*p* > 0.05) with a mean difference of 4.3. Meanwhile, magnesium nitrate and potassium nitrate were poor sources of nitrogen as they can only assist RR120 decolourisation by 11.9% and 7.9% respectively. In comparison between urea, potassium nitrate, ammonium chloride, magnesium nitrate and control, ammonium sulphate showed the best decolourisation with a significant difference (*p* < 0.001).

### 3.9. Effects of Different Ammonium Sulphate Concentrations on Decolourisation of RR120

**Figure 10:**
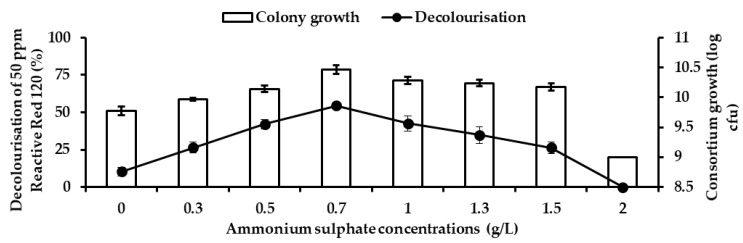
Effects of different ammonium sulphate concentration on the decolourisation of 50 ppm Reactive Red 120 by consortium JR3. Data represent mean ± SD, *n* = 3.

Figure 10 illustrates the effects of different ammonium sulphate concentrations on the decolourisation of 50 ppm RR120 and bacterial growth by consortium JR3. Decolourisation and bacterial growth steadily increased to 0.7 g/L. Above 0.7 g/L, this resulted in a gradual decrease in both the decolourisation and bacterial growth. The highest decolourisation was observed at 0.7 g/L by 54.4% with bacterial growth of 10.46 log CFU. At 1 g/L, RR120 decolourisation stood at 42.5% with bacterial growth of 10.23 log CFU, illustrating that an increase beyond 0.7 g/L resulted in lower decolourisation. Almost no decolourisation was observed at 2 g/L with 1.2% and bacterial growth at 9.2 log CFU. In comparison with control, having no nitrogen source resulted in a better decolourisation of RR120 compared to an excess of 2 g/L ammonium sulphate. A gradual decrease in decolourisation at 1, 1.3 and 1.5 g/L ammonium sulphate was observed with 42.5%, 33.1% and 21.5%, respectively. However, no significant differences were observed in bacterial growth at 1, 1.3 and 1.5 g/L. This illustrates that an increase beyond 0.7 g/L, consortium JR3 completely rely on ammonium sulphate as a sole nitrogen source.

### 3.10. Effects of pH on Decolourisation of RR120

**Figure 11:**
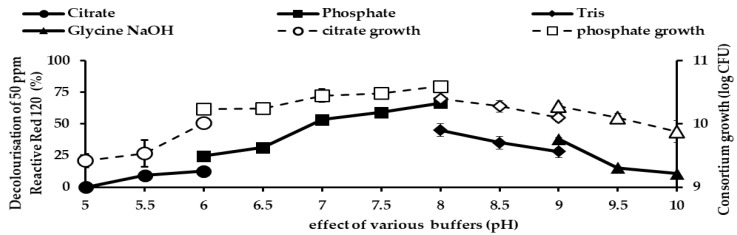
Effects of different pH buffers on the decolourisation of 50 ppm Reactive Red 120 by consortium JR3. Data represent mean ± SD, *n* = 3.

Figure 11 illustrates the effects of pH with different buffers on the decolourisation of 50 ppm RR120 and bacterial growth by consortium JR3. The highest decolourisation was observed in pH 8 aided by phosphate buffer. Phosphate buffer improved the decolourisation of RR120 by 66.5% and 53.4% at pH 7 and 8, respectively. The second-best buffer was Tris base, which results in 44.9% decolourisation at pH 8. Phosphate buffer improved decolourisation significantly compared to the rest of it, as it recorded the highest decolourisation with a mean difference of 0.84 (*p* < 0.001). Acidic pH of 5.0, 5.5 and 6.0 illustrated low decolourisation and bacterial growth. The lowest decolourisation was observed at pH 5 with 1.2% removal and bacterial growth of 9.42 log CFU, followed by pH 5.5 with a decolourisation of 9.3% and bacterial growth of 9.53 log CFU. Alkaline pH provides a relatively good decolourisation rate compared to acidic pH. Consortium JR3 showed an improvement in decolourisation of RR120 by 41.8% at pH 8.0 compared to pH 6.0, indicating that an alkaline pH was preferable for better decolourisation of RR120 than acidic conditions.

### 3.11. Effects Temperature on Decolourisation of RR120

**Figure 12:**
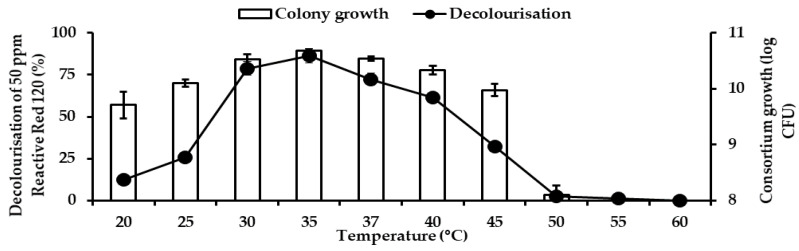
Effects of temperature on decolourisation of 50 ppm Reactive red 120 by consortium JR3. Data represent mean ± SD, *n* = 3.

Figure 12 shows the effects of temperature on RR120 decolourisation and colony growth by consortium JR3. The optimum temperature demonstrated by this consortium JR3 in decolourisation was at 35 °C. It improved decolourisation by 86.4% with a colony growth of 10.67 log CFU and 30 °C was the second-best by 78.8% decolourisation with a colony growth of 10.53 log CFU, followed by 37 °C with RR120 decolourisation by 72.2% with a colony growth of 10.51 log CFU. Meanwhile, temperatures of 25, 40 and 45 °C demonstrated decolourisations of 25.9%, 61.3% and 32.1%, respectively. Almost no colony growth and decolourisation were observed at 50 °C, where else 55 and 60 °C inhibited decolourisation completely by consortium JR3. This illustrates that increasing temperature beyond 37 °C significantly decreases colony growth, thus resulting in poor RR120 decolourisation.

### 3.12. Effects of Different RR120 Concentration on Decolourisation

**Figure 13:**
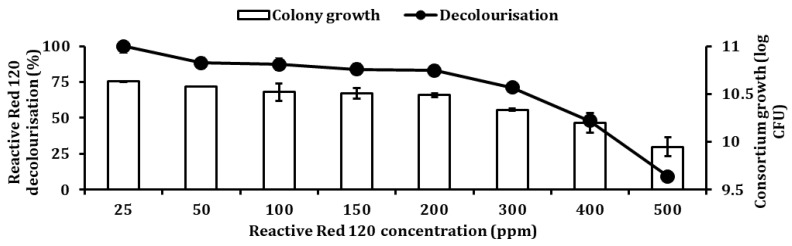
Effects of different Reactive Red 120 concentration on decolourisation by consortium JR3. Data represent mean ± SD, *n* = 3.

Figure 13 demonstrates the effects of different RR120 concentrations on its decolourisation and colony growth rate by consortium JR3. A 25 ppm of RR120 illustrated the highest decolourisation, which stood 99.3% with bacterial growth of 10.63 log CFU. Followed by 50 ppm with 88.2% decolourisation with 10.58 log CFU colony growth, 100 ppm with 87.3% decolourisation with 10.52 log CFU, 150 ppm with 83.8% decolourisation with 10.51 log CFU and 200 ppm with 82.9% decolourisation with 10.49 log CFU colony growth. Based on ANOVA, there was no significant difference (*p* > 0.05) in decolourisation from 50 to 200 ppm. This implies that concentrations ranging from 50 to 200 ppm will result in similar decolourisation rate at 24 h mark. This shows that consortium JR3 was able to decolourise 200 ppm RR120 at the same efficiency level as 50 ppm. Lowest decolourisation was observed at 500 ppm with a rate of 9.2%, which had almost reduced its decolourisation capability by 10 times compared to 25 ppm.

### 3.13. Plackett-Burman Design

The Plackett-Burman design used to screen for significant factors affecting decolourisation by consortium JR3 resulted in RR120 decolourisation levels from 36.7% to 85.71% in 12 runs. The highest decolourisation was obtained at run 1 with the following setup: 0.3 g/L ammonium sulphate, pH of 8.5, 31 °C and RR120 concentrations of 150 ppm. Meanwhile, the lowest decolourisation was observed at 0.3 g/L ammonium sulphate, pH 8.5, 40 °C and RR120 concentration of 300 ppm (Table 8).

ANOVA indicated that ammonia sulphate concentration (A), temperature (C), and RR120 concentration (D) were found to be significant (*p* < 0.05), meanwhile pH (B) was found to be insignificant (*p* > 0.05) in model terms (Table 9). However, pH interactions with ammonia sulphate concentration (AB), temperature (BC) and RR120 concentration (BD), the model results were significant (*p* < 0.05). Therefore, in this stipulation, all four factors were considered as significant model provisions in yielding a positive interaction. The analysis of variance of the quadratic regression model illustrated that the model was highly significant as the evidence from the *F*-test had a low probability value (*F* value = 428.70). Also, the analysis suggests that the “Predicted R-squared” value of 0.9972 is in reasonable agreement with the “Adjusted R-squared” of 0.9329 clarifying the impact of the model. Hence, all four factors were used in constructing a central composite design.

### 3.14. Central Composite Design

The central composite design was used to study the interaction between four significant factors in RR120 decolourisation by consortium JR3. The highest decolourisation was observed to be at 89.9%, while the lowest was observed at 12.5% in Table 10. The following conditions, 0.65 g/L ammonium sulphate, pH 7, 37 °C and 75 ppm RR120, aided the highest decolourisation; meanwhile, 0.65 g/L ammonium sulphate, pH 7, 19 °C and 225 ppm RR120 results in the lowest dye removal. ANOVA (Table 11) illustrates that the model was highly significant (*p* < 0.0001) and reproducible.

**Figure 14:**
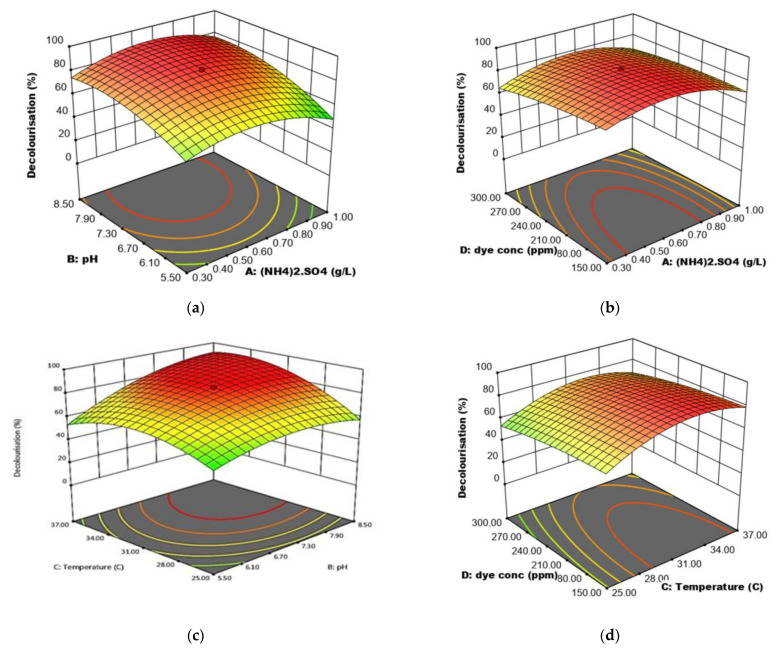
Response surface 3D contour showing (**a**) interaction between pH and ammonium sulphate concentration, (**b**) interaction between RR120 and ammonium sulphate concentration, (**c**) interaction between temperature and pH, (**d**) interaction between RR120 concentration and temperature.

Figure 14a explains the effects of ammonium sulphate concentration and pH on the decolourisation of RR120 while maintaining the concentration of RR120 at 225 ppm and temperature at 31 °C. The high percentage of RR120 decolourisation was found to occur within nitrogen concentrations (0.48–0.82 g/L) and pH range (7.0–8.5) as stated. When the pH was increased to 8.5, Consortium JR3 was able to decolourise up to 76.58% RR120 when ammonium sulphate was maintained at 0.3 g/L. An increase of 28% decolourisation ability, due to these strains’ ability to maintain better homeostasis in alkaline conditions. However, when the nitrogen source is given in excess, the decolourisation rate dropped significantly no matter in pH 5.5 or 8.5. This is due to the fact that the Consortium JR3 completely uses ammonia sulphate as the sole nitrogen source, thus leaving incomplete mineralisation of RR120. This leads to improper cleavage of the azo bond, therefore the dye loses less colour as the chromosphere remains intact.

Figure 14b demonstrates the effects of RR120 concentration and ammonium sulphate concentration while keeping a temperature of 31 °C and a pH of 6.5 at constant. The highest decolourisation of RR120 was observed at 85.2% when RR120 concentration and ammonium sulphate concentration was at 225 ppm and 0.6 g/L, respectively. Reducing ammonium sulphate concentration has significantly improved RR120 decolourisation in which Consortium JR3 was able to decolourise 20% more 150 ppm RR120 at 0.3 g/L ammonium sulphate compared to 1 g/L, in which it was able to decolourise only 55% at 1 g/L. However, when RR120 concentration increased to 300 ppm, decolourisation remained at 60 ± 1.2% when ammonium sulphate stood at 0.3 and 1.0 g/L. Decolourisation only increased significantly when ammonium sulphate was maintained at 0.65 g/L, regardless of the azo dye concentration.

Figure 14c explains the effect of pH and temperature on the decolourisation of RR120 while maintaining RR120 concentration at 225 ppm and ammonium sulphate concentration at 0.65 g/L. The interaction between temperature and pH illustrates that at 31 °C and pH 7.75, maximal decolourisation of RR120 at 91.31% was observed. In this term, the effect of pH has outweighed the effect of temperature. At pH 5.5, decolourisation of RR120 almost reduced its efficiency by half. This is because in pH 5.5, when the temperature at 25 °C and 37 °C, decolourisation was observed at 50.13% and 53.35% respectively. Based on ANOVA, when the temperature ranges from 25 °C to 37 °C, decolourisation remained insignificant in pH 5.5. Where else, when pH was increased to 8.5, huge differences in the decolourisation of RR120 was observed. As such, when pH remained at 8.5, decolourisation of RR120 was observed at 57.8% at a given temperature of 25 °C. However, when the temperature was raised to 37 °C, an increase of 34.12% in decolourisation capability was observed. This suggests that, when ammonium sulphate concentration remains at 0.65 g/L, the effect of pH is far greater than temperature. When pH remains at 5.5, decolourisation was observed to be lower regardless of the temperature.

Figure 14d illustrates the effect of temperature and RR120 concentration while maintaining ammonium sulphate concentration at 0.65 g/L and pH of 6.5. The result shows that, as temperature and RR 120 concentration increased, decolourisation increased until the optimum condition was obtained. Low decolourisation of RR120 was observed at 25 °C regardless of the dye concentration. As such, the least decolourisation was recorded at a temperature of 25 °C and a dye concentration of 300 ppm, with 53.01%. An increase in dye concentration to 300 ppm significantly reduces decolourisation. As temperature increases, the rate of enzymatic activity increases proportionally to a certain point. This trend can be seen when an increase in temperature improves decolourisation by 21.5% from 25 °C to 37 °C at 150 ppm RR120. However, when dye concentration increased to 300 ppm, decolourisation remained low despite increasing the temperature. This suggests that an increase in dye concentration from 150 ppm, at pH 6.5 and ammonium sulphate concentration of 0.65 g/L, decolourisation remains low regardless of the temperature.

### 3.15. Validation of CCD Prediction

According to the numerical solution provided by CCD, the best factors for maximum RR120 decolourisation was an ammonium sulphate concentration of 0.645 g/L, pH of 8.293 and a temperature of 34.532 °C, which yields a theoretical 93.66% decolourisation of 200.058 ppm RR120 concentration. A confirmation experiment was organised with suggested optimal parameters from CCD to verify the result. Consortium JR3 was able to decolourise 92.79% of 195.058 ppm RR120 in a period of 24 h, which illustrated high similarity with the predicted decolourisation rate. Moreover, Tukey analysis showed no significant difference (*p* > 0.05) between the CCD predicted result and the validation result. This is in line with the model prediction.

## 4. Discussion

A dye obtains its colour through chromophores and auxochromes. By altering the overall electron energy system, the dye loses its colour, hence decolourisation occurs [4,32]. However, the term decolourisation is often confused with degradation among researchers. Decolourisation only breaks down the electron system, meanwhile, degradation breaks down the electron system and the resulting compounds are used as energy or nitrogen source [37]. This has led to various researchers using different media compositions for isolating dye degrading bacteria. Certain media promotes decolourisation while others inhibit degradation.

This is the first study comparing the role of several media in the isolation method and their influence on RR120 decolourisation. Only consortium JR3 was able to decolourise RR120 maximally without the aid of additional carbon sources and co-substrates as compared to consortia JR1 and JR2. This is the first study to report a combination of *Pseudomonas aeruginosa*, *Serratia marcescens* and *Enterobacter* sp. that were able to degrade and consume RR120 as the sole carbon source without the aid of co-substrate. Even though the observed decolourisation was lower than with the addition of extra carbon sources, the consortium can completely mineralise the dye. *Bacillus lentus* B1377 [38] was able to achieve complete mineralisation of RR120 when supplemented with NB, on other hand, *Pseudomonas gualiconensis* [39] was reported to produced toxic metabolite in similar condition. Most of RR120 decolouriser needs glucose/yeast extract more than 3 g/L to achieve significant decolourisation [16,26,30,39,40]. Consortium JR3 was able to do so with the least amount of co-substrate.

The process of acclimatisation is more beneficial as it improved the decolourisation capability of JR3. This can be illustrated in MSM 4 when JR3 outperformed JR2 in decolourisation term. Meanwhile, in MSM 3, supplementing a yeast concentration of 1 g/L significantly improved decolourisation ability of JR3 followed by JR2. However, no noticeable decolourisation was observed in JR2 and JR3 when RR120 was supplemented with NB as compared to JR1. Under this condition, RR120 is rather being absorbed by the consortium than being degraded (data not shown). A similar effect was also observed in MSM 2 and MSM 5. Both consortia grow on readily available carbon source and the dye are not being consumed.

The results are in agreement with Brilon et al. [34]. When no glucose and yeast extract was given to the culture medium, degradation of naphthalenesulfonic acids was extremely poor in *Pseudomonas* sp. A3 [34]. When yeast extract was introduced at a concentration of 0.05% (*w*/*v*), degradation is improved. They also illustrated that *Pseudomonas* sp. C22 that is unable to grow on naphthalenesulfonic acids, is able to grow when the same concentration of yeast extract is introduced [34]. Thus, it could be summarised that the presence of co-substrate significantly improves decolourisation of RR120 consortium JR3, and without the addition of co-substrate, decolourisation appears to be slow. Consortium JR3 was chosen as the best consortium to decolourise RR120 as it requires less co-substrate and is able to consume RR120 without the presence of any additional carbon source, which is extremely rare in the field of azo dye bioremediation.

Similarly, when co-substrate is not available, the presence of trace elements improves the decolourisation condition. The availability of these nutrients for microbes responsible for aerobic digestion and substrate toxicity. The most important trace elements in micronutrients mostly involved in aerobic digestion efficiency are cobalt, nickel, molybdenum, zinc and magnesium [41]. Molybdenum functions as cofactors for various enzymes, nickel is needed for the synthesis of coenzymes, zinc is known for stimulating cell growth, iron acts as an electron acceptor, calcium is needed for membrane permeability and both copper and cobalt for metallic enzyme activator [42].

The role of yeast extract is considered essential for the regeneration of NADH, which acts as the electron donor for the reduction of azo bonds [43]. Yeast extract comprised components such as riboflavin, thiamine and pyridoxine, which enhance and improve bacterial growth and azo dye decolourising activity. However, the right amount of yeast extract to be used in dye decolourisation remains debatable among researchers. Some researchers found that increasing yeast extract concentration enhances decolourisation rate while others report the opposite [12,44]. Consortium JR3 falls into the latter category. This behaviour might be attributed to the fact that bacterial strains consume yeast extract as readily available carbon and nitrogen source for their growth instead of targeting the destruction of azo dye bond. The role of the electron donor taken by RR120 is a result of the fortuitous process [45]. When this occurs, RR120 are not broken down properly, thus the colour loss will be lower.

Eskandari et al. [12] reported that ammonium dihydrogen phosphate improved Reactive Blue 5 decolourisation better compared to peptone and yeast extract in both cold-adapted and mesophilic consortium. Organic nitrogen metabolism is crucial for the regeneration of NADH, as it plays an important role as an electron donor [46]. Organic nitrogen sources, such as peptone, yeast and beef extract, have been found effective in improving dye removal rate in a mesophilic microorganism [30]. However, excess of this nitrogen source will result in the dye not being properly mineralised, as bacteria start consuming available carbon to grow [37]. This finding is consistent with the result of Fouda et al. [47], who reported that ammonium sulphate significantly improved decolourisation of Disperse Blue, Disperse Yellow and Reactive Red Syozol compared to rest of the nitrogen sources in *Pseudomonas stutzeri* SB13.

Additionally, dye decolourisation does not indicate that resulting metabolites are less toxic and safe compared to that parent compounds [48]. An assessment on resulting metabolite should be done to determine the safety of remediated dyes. Seed germination percentage, seedling survival and seedling height have been taken as important criteria to assess plant response to specific pollutants [7,35,39]. The presence of azo dye could directly affect the role of chlorophyllase and abscisic acid (ABA), therefore impeding the growth of the plant [49]. The severity of dye toxic effect depends on metabolite(s) produced during the decolourisation process. Rawat et al. [4] found that treating Acid Orange 7 decolourised metabolites significantly reduced the overall shoot biomass of *Allium cepa* because of poor uptake and transfer of the nutrient. These metabolites were later detected to be aniline, 1-amino-2-naphthol, naphthalene, and phenyl diazene, the consequences of improver azo dye decolourisation by halophilic microbial consortium. On the other hand, Nouren et al. [50] reported a better improvement in *Zea mays* seed germination, shoot and root length when exposed to treated Direct Yellow 4 compared to untreated samples, which result in a 50% inhibition rate. Similarly, Santana et al. [51] also observed an increase in lettuce and clove seed germination when exposed to a treated sample of Reactive Red 195 compared to the parent compound as a result of complete mineralisation of the azo dye. The finding of Santana et al. [51] also supported by the work of Roy et al. [52] who reported significant improvement in chickpea seed germination when exposed to a treated sample of Reactive Yellow. Therefore, it can be summarised that a proper decolourisation of a dye compound will result in total mineralisation, which significantly reduces the toxicity of the dye.

The nature of the role of pH on dye decolourisation is influenced by the location of the strain obtained. Bacteria that are isolated from the acidic environment are able to degrade dye better in pH less than 7 [53], meanwhile, those from alkaline environment perform exceptionally well in pH more than 7 [24]. Previous repeated exposure to acidic or alkaline environment allowed the azoreductase enzyme to acclimatise to their appropriate environment pH; thus, having a distinct affinity towards substrate in different pH environments. Since consortium JR3 was isolated from a contaminated site near to a fabric manufacturing factory, frequent exposure to alkaline effluents from washing activity (detergent and soap) allowed the enzyme to acclimatize to an alkaline condition. Similarly, *Halomonas* sp. isolated from textile wash water showed an optimal pH of 9–12 for maximal removal of Reactive Red 152 [54]. Meanwhile, *Enterococcus casseliflavus* RDB4 had an optimal pH of 7 and better decolourisation of Reactive Red 195 is observed under alkaline conditions compared to acidic medium [55]. *Pseudomonas aeruginosa* strain HF5 was able to decolourise 90.9% and 82.5% RR120 at pH values of 7.5 and 8.5 at 24 h, respectively [30]. In most of the reactive red decolourisation works occurring under aerobic alkaline conditions, the detected metabolite is 6-hydroxy cyclohexa 2, 4-dienone, which is a very common desulphonation reaction [38,56].

Temperature influences bacterial growth rate and enzymatic reaction. In this study, the optimum temperature was 35 °C. In other studies, the optimum temperature for RR120 removal was 35 °C in *Shewanella haliotis* RDB1 [37]. No decolourisation was observed beyond 55 °C in our result, similar to *Shewanella haliotis* RDB1. Consortium RV2 was able to decolourise 87.05% of 100 ppm Reactive Red 31 at the optimal temperature of 37 °C within 12 h under microaerophilic conditions [57].

The ability of *Pseudomonas* sp. SUK1 to decolourise RR120 has been reported [58]. The strain was able to achieve 97% decolourisation of 150 ppm RR120 at 100 h. However, since nutrient broth and static conditions were used for *Pseudomonas* sp. SUK1 decolourisation conditions, resulting in toxic metabolites needing to undergo radiation treatment to breakdown those by-products. Meanwhile, *Pseudomonas guariconensis* was able to decolourise a maximum of 48% RR120 at 70 h [39]. Even in fungal species, decolourisation of RR120 was reported at 100 ppm with the aid of glucose [59], whereby consortium JR3 was able to decolourise even better without the need for glucose and still achieved significant decolourisation at 400 ppm.

In terms of decolourisation efficiency, RSM resulted in a better decolourisation capability compared to the one-factor-at-a-timeresult. This study also shows similar improvement observed in other degradation optimisation works [60,61]. Although both OFAT and RSM showed almost similar values, minor changes in RSM value have significantly improved the decolourisation rate of RR120.

## 5. Conclusions

This study illustrates that a long period of slow acclimation process led to the isolation of bacterial strains capable of utilising RR120 as a sole carbon source without the presence of additional carbon or co-substate. The presence of yeast extract significantly improves decolourisation rate in azo dyes; however, the concentration should be limited to a smaller amount as an increased available carbon source will lead to the decolourisation of azo dyes as a non-assimilatory process and this will not reduce the toxicity of the existing dye. This is the first study to successfully compare the role of isolation method in influencing decolourisation ability based on different media composition. The use of nutrient broth should be limited as the decolourisation under this medium produces toxic metabolites in which significant inhibition of *Vigna radiata* germination, shoot and root length was observed.

## Figures and Tables

**Table 1:** GPS location list of sampling area sites.

Location	GPS
Sungai Juru, Pulau Pinang	5°20′47.3″ N 100°25′05.1″ E
Sungai Juru, Pulau Pinang	5°20′46.7″ N 100°25′04.5″ E
Sungai Juru, Pulau Pinang	5°20′42.8″ N 100°25′06.7″ E
Sungai Juru, Pulau Pinang	5°20′38.1″ N 100°25′10.1″ E

**Table 2:** Different media composition used for finding suitable dye decolourisation medium.

MSM	Sample	Media Composition (g/L)	Trace Element (mg/L)	Dye	Reference
1.	Textile waste water	NaCl (1.0) CaCl_2_.2H_2_O (0.1) MgSO_4_.7H_2_O (0.5) K_2_HPO_4_ (1.0) Yeast extract (3.0)		Reactive Red 120	[30]
2.	Textile waste water	NaCl (1.0) CaCl_2_.2H_2_O (0.1) MgSO_4_.7H_2_O (0.5) KH_2_PO_4_ (1.0) NH_4_.SO_4_ (2.0) Na_2_HPO_4_ (1.0) yeast extract (5.0)	-	Direct Red 81, Acid Red 88, Reactive Black 5	[31]
3.	Waste water	NaCl (1.0) CaCl_2_.2H_2_0 (0.1) MgSO_4_.7H_2_O (0.5) KH_2_PO_4_ (1.0) Na_2_HPO_4_.2H_2_O (1.0) Yeast extract (1.0)	-	Reactive Red 120	[26,32]
5.	Waste water	Na_2_HPO_4_.2H_2_O (31.7) KH_2_PO_4_ (3) KH_4_Cl (0.5) NaCl (0.5) MgSO_4_.7H_2_O (0.12) Glucose (8.0)	CaCl_2_ (4.0)	Reactive Red 180	[33]
4.	Sewage	Na_2_HPO_4_.2H_2_O (12.0) KH_2_PO_4_ (2.0) NH_4_NO_3_ (0.50) MgCl_2_.6H_2_O (0.10) Ca (NO_3_)_2_.4H_2_O (0.050) FeCl_2_.4H_2_O(0.0075)	ZnCl _3_ (50) MnCl_2_.4H_2_O (30) CoCl_2_.6H_2_O (200) NiC_l2_.6H_2_O (20) Na_2_MoO_4_.2H_2_O (30) H_3_BO_3_ (300) CuCl_2_.2H_2_O (10)	Naphthalene sulfonic Acid	[34]

**Table 3:** Plackett-Burman design for tested factors.

Factor	Parameter	Unit	Low Level (−1)	High Level (+1)
A	Ammonium sulphate	g/L	0.3	1.0
B	pH		6.5	8.5
C	Temperature	°C	31	40
D	RR120	ppm	150	300

**Table 4:** Central composite design for tested factors.

Factor	Parameter	Unit	Low Level (−1)	High Level (+1)
A	Ammonium sulphate	g/L	0	1.35
B	pH		5.5	9.5
C	Temperature	°C	19	43
D	RR120	ppm	75	375

**Table 5:** Morphology and biochemical properties of the different isolates (+), strain positive; (-), strain negative.

	Isolate	Name	Gram Staining	Oxidase	Catalase	VP	Nitrate Reduction	Citrate	Indole Production	Urease	Methyl Red
Consortium JR1	JR1-1	*Pseudomonas* sp. MM02	-	-	-	-	+	+	-	-	-
	JR1-2	*Pseudomonas* sp. MM03	-	-	-	-	+	+	-	-	-
	JR1-3	*Serratia* sp. MM07	-	-	-	+	+	+	-	+	-
	JR1-4	*Vibrio* sp. MM09	-	+	+	+	+	-	-	-	-
Consortium JR 2	JR2-1	*Serratia* sp. MM08	-	-	-	+	+	+	-	+	-
	JR2-2	*E. cloacae* MM04	-	-	+	+	+	+	-	+	-
Consortium JR 3	JR3-1	*Enterobacter* sp. MM05	-	-	+	+	+	+	-	-	-
	JR3-2	*P. aeruginosa* MM01	-	+	+	-	+	+	-	-	-
	JR3-3	*S. marcescens* MM06	-	-	+	+	-	+	-	+	-

**Table 6:** Identified isolates name based on phylogenetic analysis with registered accession number in National Center for Biotechnology Information

Isolate	Strain Name	Ascension No.
JR1-1	*Pseudomonas* sp. MM02	MW024071
JR1-2	*Pseudomonas* sp. MM03	MW024072
JR1-3	*Serratia* sp. MM07	MW031903
JR1-4	*Vibrio* sp. MM09	MW227497
JR2-1	*Serratia* sp. MM08	MW031904
JR2-2	*Enterobacter cloacae* MM04	MW025258
JR3-1	*Enterobacter* sp. MM05	MW031860
JR3-2	*Pseudomonas aeruginosa* MM01	MW024070
JR3-3	*Serratia marcescens* MM06	MW031902

**Table 7:** Phytotoxicity study of Reactive Red 120 and its metabolite formed after decolourisation values are the mean of the germinated seeds of the three experiments, SD (±), standard deviation.

Condition	Removal (%)	Seed Germination (%)	Root Length (cm)	Shoot Length (cm)
No dye	-	100 ± 0	5.77 ± 0.06	10.37 ± 0.67
50 ppm RR120	0 ± 0.01	63.3 ± 2.52	4.23 ± 0.15	7.77 ± 0.55
JR1 + NB	67.6 ± 4.78	21.3 ± 2.64	1.23 ± 0.59	4.87 ± 0.25
JR1 + MSM1	2.1 ± 0.24	65.3 ± 0.93	4.3 ± 0.26	7.83 ± 0.32
JR1 + MSM2	29.0 ± 1.3	48.7 ± 1.25	3.97 ± 0.12	5.33 ± 0.35
JR1 + MSM 3	2.3 ± 0.01	68 ± 2	4.39 ± 0.01	7.70 ± 0.01
JR1 + MSM 4	0.0 ± 0.01	62.3 ± 3.95	4.27 ± 0.06	7.70 ± 0.53
JR1 + MSM 5	35.2 ± 2.12	25.7 ± 2.52	2.3 ± 0.26	5.10 ± 0.36
JR2 + NB	3.2 ± 0.01	62.7 ± 3.66	4.4 ± 0.17	7.80 ± 1.2
JR2 + MSM 1	11.7 ± 0.02	67.3 ± 2	4.07 ± 0.15	8.10 ± 0.17
JR2 + MSM 2	1.7 ± 0.65	65 ± 0.2	4.40 ± 0.1	7.80 ± 1.05
JR2 + MSM 3	18.7 ± 1.2	69.3 ± 1.51	4.77 ± 0.06	8.13 ± 0.32
JR2 + MSM 4	5.9 ± 0.12	69.7 ± 1.15	4.5 ± 0.3	7.80 ± 0.2
JR2 + MSM 5	0.3 ± 0.01	63.3 ± 1.8	4.2 ± 0.1	7.73 ± 0.85
JR3 + NB	2.3 ± 0.1	66 ± 0.2	4.27 ± 0.06	7.73 ± 0.25
JR3 + MSM 1	8.3 ± 0.8	73.3 ± 0.45	4.33 ± 0.38	8.00 ± 0.1
JR3 + MSM 2	7.0 ± 1.2	71.3 ± 0.06	4.43 ± 0.06	7.87 ± 1
JR3 + MSM 3	34.2 ± 2.5	81.7 ± 0.95	5.17 ± 0.6	9.10 ± 0.69
JR3 + MSM 4	10.8 ± 0.53	75 ± 3.61	4.57 ± 0.21	8.17 ± 0.45
JR3 + MSM 5	0.8 ± 0.2	66 ± 1	4.2 ± 0.36	7.90 ± 1

**Table 8:** Experimental design and results of Plackett-Burman on Reactive Red 120 decolourisation by consortium JR3.

Run	A	B	C	D	Actual Value	Predicted Value
1	0.30	8.5	31	150	85.71	86.00
2	0.30	6.5	31	150	84.41	83.83
3	0.30	6.5	40	300	56.39	56.68
4	1.00	6.5	31	150	83.02	83.31
5	1.00	8.5	40	150	48.05	48.34
6	0.30	6.5	31	300	51.51	51.80
7	1.00	8.5	31	300	80.69	80.17
8	0.30	8.5	40	150	44.43	43.85
9	1.00	6.5	40	300	45.98	45.40
10	1.00	6.5	40	150	62.89	63.18
11	1.00	8.5	31	300	79.94	80.17
12	0.30	8.5	40	300	36.74	37.03

A: ammonia sulphate concentration (g/L), B: pH, C: temperature (°C), D: RR120 concentration (ppm).

**Table 9:** Analysis of factors for Plackett-Burman on RR120 decolourisation by consortium JR3.

Source	Sum of Square	dF	Mean Square	*F* Value	Prob > F	
Modal	3695.08	9	410.56	428.70	0.0023	significant
A	7.31	1	7.31	7.63	0.0199	
B	1.59	1	1.59	1.66	0.3262	
C	1249.32	1	1249.32	1304.50	0.0008	
D	700.87	1	700.87	731.82	0.0014	
AB	115.42	1	115.42	120.52	0.0082	
AC	53.72	1	53.72	56.10	0.0174	
BC	581.87	1	581.87	607.57	0.0016	
BD	55.74	1	55.74	58.20	0.0168	
CD	94.32	1	94.32	98.49	0.0100	
Residual	1.92	2	0.96			
Lack of Fit	1.63	1	1.63	5.81	0.2504	Not significant
Pure Error	0.28	1	0.28			
Cor Total	3696.99	11				
Std. dev.	0.98		R-squared		0.9995	
Mean	63.31		Adj R-squared		0.9972	
C.V	1.55		Pred R-squared		0.9329	
PRESS	248.19		Adeq Precision		54.816	

A: ammonia sulphate concentration (g/L), B: pH, C: temperature (°C), D: RR120 concentration (ppm).

**Table 10:** Experimental design and results of central composite design on Reactive Red 120 decolourisation by consortium JR3.

Run	A	B	C	B	Actual Value	Predicted Value
1	0.30	8.50	25.00	300.00	38.62	37.45
2	0.65	7.00	43.00	225.00	50.2	49.76
3	1.00	8.50	25.00	300.00	45.21	45.47
4	0.30	8.50	25.00	150.00	46.5	45.36
5	0.30	8.50	37.00	150.00	79.3	79.64
6	0.65	10.00	31.00	225.00	73.3	73.62
7	1.00	5.50	25.00	150.00	35	32.55
8	0.65	7.00	31.00	375.00	64.5	66.87
9	1.00	8.50	25.00	150.00	42.2	43.32
10	0.65	7.00	31.00	225.00	85.2	83.7
11	0.30	5.50	25.00	150.00	47.2	48.42
12	0.30	8.50	37.00	300.00	72	71.03
13	0.65	7.00	31.00	225.00	85.2	85.9
14	0.65	7.00	31.00	225.00	85.2	85.9
15	0.65	7.00	31.00	225.00	85.2	85.9
16	0.65	7.00	31.00	225.00	85.2	85.9
17	1.00	5.50	25.00	300.00	27.12	27.19
18	0.65	7.00	31.00	225.00	85.2	85.9
19	1.00	5.50	37.00	300.00	29	29.56
20	1.00	8.50	37.00	150.00	75.2	76.76
21	0.30	5.50	25.00	300.00	32.3	30.16
22	1.00	5.50	37.00	150.00	36.9	38.48
23	0.30	5.50	37.00	150.00	53.2	52.36
24	0.65	7.00	31.00	75.00	89.9	83.7
25	1.35	7.00	31.00	225.00	35	32.55
26	1.00	8.50	37.00	300.00	79	78.19
27	0.30	5.50	37.00	300.00	34.1	33.39
28	0.65	4.00	31.00	225.00	28.2	28.05
29	−0.05	7.00	31.00	225.00	35.8	38.42
30	0.65	7.00	19.00	225.00	12.5	13.11

A: ammonia sulphate concentration (g/L), B: pH, C: temperature (°C), D: RR120 concentration (ppm).

**Table 11:** Analysis of factors for central composite design on Reactive red 120 decolourisation by consortium JR3.

Source	Sum of Squares	dF	Mean Square	*F* Value	Prob > F	
Model	15774.23	14	1126.73	398.84	<0.0001	Significant
A	51.6	1	51.6	18.26	0.0007	
B	3114.71	1	3114.71	1102.55	<0.0001	
C	2015.75	1	2015.75	713.54	<0.0001	
D	424.62	1	424.62	150.31	<0.0001	
A^2^	4236.93	1	4236.93	1499.79	<0.0001	
B^2^	2024.44	1	2024.44	716.61	<0.0001	
C^2^	4955.37	1	4955.37	1754.1	<0.0001	
D^2^	168.51	1	168.51	59.65	<0.0001	
AB	120.84	1	120.84	42.77	<0.0001	
AC	0.73	1	0.73	0.26	0.6194	
AD	101.05	1	101.05	35.77	<0.0001	
BC	920.97	1	920.97	326.01	<0.0001	
BD	107.17	1	107.17	37.94	<0.0001	
CD	0.51	1	0.51	0.18	0.6776	
Residual	42.38					
Lack of fit	42.38		4.238	4.01	0.0322	Not significant
Pure Error	0.000					
Cor total	15816.60					
Std dev.	1.68		R-Squared		0.9973	
Mean	55.65		Adj R-Squared		0.9948	
C.V	3.02		Pred R-Squared		0.9846	
PRESS	244.08		Adeq Precision		60.660	

A: ammonia sulphate concentration (g/L), B: pH, C: temperature (°C), D: RR120 concentration (ppm).

## Data Availability

The data presented in this study are openly available in FigShare.com (accessed on 10 August 2020), https://doi.org/10.6084/m9.figshare.14128694.v1.
